# Protein disulfide isomerase-mediated *S*-nitrosylation facilitates surface expression of P2X7 receptor following status epilepticus

**DOI:** 10.1186/s12974-020-02058-y

**Published:** 2021-01-06

**Authors:** Duk-Shin Lee, Ji-Eun Kim

**Affiliations:** Department of Anatomy and Neurobiology, Institute of Epilepsy Research, College of Medicine, Hallym University, Kangwon-Do, Chuncheon, 24252 South Korea

**Keywords:** Apoptosis, Astrocyte, L-NAME, Microglia, NF-κB, Nitric oxide, Redox, Seizure

## Abstract

**Background:**

P2X7 receptor (P2X7R) is an ATP-gated nonselective cationic channel playing important roles in a variety of physiological functions, including inflammation, and apoptotic or necrotic cell death. An extracellular domain has ten cysteine residues forming five intrasubunit disulfide bonds, which are needed for the P2X7R trafficking to the cell surface and the recognition of surface epitopes of apoptotic cells and bacteria. However, the underlying mechanisms of redox/*S*-nitrosylation of cysteine residues on P2X7R and its role in P2X7R-mediated post-status epilepticus (SE, a prolonged seizure activity) events remain to be answered.

**Methods:**

Rats were given pilocarpine (380 mg/kg i.p.) to induce SE. Animals were intracerebroventricularly infused *N*^ω^-nitro-l-arginine methyl ester hydrochloride (L-NAME, a NOS inhibitor) 3 days before SE, or protein disulfide isomerase (PDI) siRNA 1 day after SE using an osmotic pump. Thereafter, we performed Western blot, co-immunoprecipitation, membrane fraction, measurement of *S*-nitrosylated (SNO)-thiol and total thiol, Fluoro-Jade B staining, immunohistochemistry, and TUNEL staining.

**Results:**

SE increased *S*-nitrosylation ratio of P2X7R and the PDI-P2X7R bindings, which were abolished by L-NAME and PDI knockdown. In addition, both L-NAME and PDI siRNA attenuated SE-induced microglial activation and astroglial apoptosis. L-NAME and PDI siRNA also ameliorated the increased P2X7R surface expression induced by SE.

**Conclusions:**

These findings suggest that PDI-mediated redox/*S*-nitrosylation may facilitate the trafficking of P2X7R, which promotes microglial activation and astroglial apoptosis following SE. Therefore, our findings suggest that PDI-mediated regulations of dynamic redox status and *S*-nitrosylation of P2X7R may be a critical mechanism in the neuroinflammation and astroglial death following SE.

## Background

Nitric oxide (NO), a gaseous second messenger, reacts with sulfhydryl (-SH, thiol) groups of proteins and leads to the formation of *S*-nitrosothiols (-SNO), so-called *S*-nitrosylation [1, 2]. *S*-nitrosylation regulates activities of target proteins via modulation of active site cysteine (C) residues or via allosteric changes of protein structures [3, 4]. Status epilepticus (SE, a prolonged seizure activity) leads to activate signaling cascades for the NO synthesis and increases NO metabolites [5–8]. However, SE-induced NO synthesis is not turned off by seizure termination, although it is initiated by SE on-set. In addition, *N*^ω^-nitro-l-arginine methyl ester hydrochloride (L-NAME, a NOS inhibitor) prevents this prolonged NO production without affecting seizure activity [7, 8]. Therefore, it is likely that prolonged NO synthesis may be involved in the diverse post-SE events rather than the generation of seizure activity, which have been elusive.

P2X7 receptor (P2X7R) is an ATP-gated nonselective cationic channel playing important roles in various physiological functions, including Ca^2+^ signaling, neurotransmitter and hormone release, inflammation, and apoptotic or necrotic cell death [9–11]. Furthermore, P2X7R regulates neuronal excitability [12], astroglial apoptosis/autophagy [13, 14], nuclear factor-κB (NF-κB) activity [15], microglial activation [16], and leukocyte infiltrations [17] following pilocarpine-induced SE. P2X7R is composed of a large extracellular domain, two transmembrane domains, and cytoplasmic N- and C-termini [18]. An extracellular domain has ten cysteine residues forming five intrasubunit disulfide bonds (SS1–SS5) [19], which are needed for the trafficking of P2X7R to the cell surface [20] and the recognition of surface epitopes of apoptotic cells and bacteria [21]. However, the underlying mechanisms of redox/*S*-nitrosylation of these disulfide bonds on P2X7R and its role in P2X7R-mediated post-SE events remain to be answered.

Protein disulfide isomerase (PDI) is an abundant oxidoreductase with chaperone activity that is predominantly expressed in the endoplasmic reticulum (ER) and cell surface. PDI has a thioredoxin (Trx)-like Cys-Xaa-Xaa-Cys (CXXC) catalytic domain, which breaks and reforms disulfide bonds (disulfide isomerization) [7, 22–25]. In addition, PDI can transfer NO onto SH groups across cell membranes, which mediates *S*-nitrosylation of various proteins [8, 25]. Thus, it is noteworthy to explore the role of PDI-mediated redox and/or *S*-nitrosylation of P2X7R in the pathophysiology induced by SE.

In the present study, therefore, we investigated whether (1) SE leads to redox/*S*-nitrosylation of P2X7R mediated by PDI; (2) these post-translational modifications of P2X7R affect post-SE events, such as neuronal death, microglial activation, reactive astrogliosis and astroglial apoptosis; or (3) PDI-mediated redox/*S*-nitrosylation regulates P2X7R trafficking following SE. Here, we demonstrate that SE increased *S*-nitrosylation ratio of P2X7R concomitant with the reduction in the amount of total thiol-P2X7R, which were abolished by L-NAME and PDI knockdown. In addition, both L-NAME and PDI siRNA attenuated SE-induced microglial activation and astroglial apoptosis. L-NAME reduced the amount of SNO-thiol on PDI and the *S*-nitrosylation ratio of PDI following SE. Both L-NAME and PDI siRNA abolished the increases in PDI-P2X7R bindings and surface expression of P2X7R induced by SE. These findings suggest that PDI-mediated *S*-nitrosylation may upregulate P2X7R surface expression, which promotes microglial activation and astroglial apoptosis following SE. Therefore, our findings suggest that PDI-mediated regulations of *S*-nitrosylation and dynamic redox status of P2X7R may be a critical mechanism in the neuroinflammation and astroglial death following SE.

## Methods

### Experimental animals and chemicals

Male Sprague-Dawley (SD) rats (7 weeks old) were used in the present study. All experimental protocols described below were approved by the Institutional Animal Care and Use Committee of Hallym University (Chuncheon, Republic of Korea), and all efforts were made to minimize animal suffering. All reagents were obtained from Sigma-Aldrich (St. Louis, MO, USA), except as noted.

### L-NAME treatment

Three days before SE induction, rats were anesthetized with isoflurane (3% induction, 1.5–2% for surgery and 1.5% maintenance in a 65:35 mixture of N_2_O:O_2_). A brain infusion kit 1 (Alzet, USA) was implanted into the right lateral ventricle (1 mm posterior; 1.5 mm lateral; 3.5 mm depth from bregma) and connected to an osmotic pump (1007D, Alzet, USA) containing vehicle (saline) or L-NAME (15 μg/μl), which were continuously infused over a 6-day period. The osmotic pump was subcutaneously placed in the interscapular region. To evaluate the seizure activity in response to pilocarpine, some animals were also implanted with a monopolar electrode (Plastics One Inc., USA) into the left dorsal hippocampus (3.8 mm posterior; 2.0 mm lateral; 2.6 mm depth from bregma). The reference electrode was placed in the posterior cranium over the cerebellum.

### SE induction

For SE induction, animals were treated with pilocarpine (380 mg/kg, single injection, i.p.) 20 min after atropine methylbromide (5 mg/kg i.p.) injection. Control animals were received an equal volume of normal saline instead of pilocarpine after the pretreatment with atropine methylbromide. In electrode-implanted animals, electroencephalogram (EEG) signals were recorded with a DAM 80 differential amplifier (0.1–3000 Hz bandpass; World Precision Instruments, USA). The data were digitized (400 Hz) and analyzed using LabChart Pro v7 (AD Instruments, Australia). Spectrograms were automatically calculated using a Hanning sliding window with 50% overlap by LabChart Pro v7 (AD Instruments, Australia). Total EEG power was normalized by the baseline power obtained from each animal. Time of SE on-set was defined as the time point showing paroxysmal depolarizing shift, which lasted more than 3 s and consisted of a rhythmic discharge between 4 and 10 Hz with amplitude of at least two times higher than the baseline EEG. After seizure onset, behavioral seizures were also measured with Racine’s score (score 1, immobility, eye closure, twitching of vibrissae, sniffing, facial clonus; score 2, head nodding associated with more severe facial clonus; score 3, clonus of one forelimb; score 4, rearing, often accompanied by bilateral forelimb clonus; and score 5, rearing with loss of balance and falling accompanied by generalized clonic seizures). Two hours after onset of SE, diazepam (Valium; Hoffman la Roche, Neuilly sur-Seine, France; 10 mg/kg, i.p.) was administered. Four rats were needed additional diazepam treatment (2–3 times) to cease SE. Three rats were not induced SE by a single pilocarpine injection, and five rats died during SE or post-SE treatment. These animals were omitted from the experimental groups. Three days after SE, animals were used for Western blot, co-immunoprecipitation, measurements of SNO-thiol or total thiol, and immunohistochemistry (see below).

### PDI knockdown

Since PDI knockdown increases the seizure threshold in response to pilocarpine [7, 8], L-NAME-untreated animals were given control siRNA or PDI siRNA 1 day after SE. siRNAs were infused using Alzet 1003D osmotic pump (Alzet, USA) over a 2-day period by the same method aforementioned. The PDI siRNA sequence used was sense, CUGCAAAACUGAAGGCAGAUU, and antisense, UCUGCCUUCAGUUUUGCAGUU. A non-silencing RNA was used as the control siRNA. AteloGene Systemic Use kit (Koken, Japan) was used for the delivery of all siRNAs, according to the manufacturer’s instructions. Three days after SE, animals were used for the studies described above.

### Western blot

Animals were sacrificed by decapitation under urethane anesthesia (1.5 g/kg, i.p.). The hippocampus was dissected out and homogenized in lysis buffer (50 mM Tris containing 50 mM 4-(2-hydroxyethyl)-1-piperazineethanesulfonic acid (pH 7.4), ethylene glycol tetraacetic acid (pH 8.0), 0.2% Tergitol type NP-40, 10 mM ethylenediaminetetraacetic acid (pH 8.0), 15 mM sodium pyrophosphate, 100 mM β-glycerophosphate, 50 mM NaF, 150 mM NaCl, 2 mM sodium orthovanadate, 1 mM phenylmethylsulfonyl fluoride, and 1 mM dithiothreitol). Total protein content was measured by BCA protein assay kit. Western blot was performed according to standard procedures. The primary antibodies were mouse anti-PDI (1:1,000, Abcam, ab2792) and rabbit anti-extracellular P2X7R IgG (1:200, Alomone labs, APR-008). The rabbit anti-β-actin (1:6000, Sigma, A5316) or rabbit anti-N-cadherin (1:4,000, Abcam, ab18203) primary antibodies were used as internal references. The signals were scanned and analyzed by ImageQuant LAS4000 system (GE health). The values of each sample were normalized with the corresponding amount of anti-β-actin (input) or N-cadherin (membrane fraction). The ratio of phosphoprotein to total protein was described as phosphorylation levels.

### Co-immunoprecipitation and membrane fraction

The tissues were lysed in radioimmune precipitation buffer (RIPA) with protease and phosphatase inhibitor cocktails (Roche Applied Sciences) and 1 mM sodium orthovanadate. After quantification of total protein concentration, equal amounts of protein (amount in 150 μg) were precipitated with the PDI antibody (10 μg/ml) and subsequently incubated with protein G sepharose at 4 °C overnight [7, 8, 22]. Beads were collected, eluted in sample buffer, and boiled at 95 °C for 5 min. The elution step was performed under the same conditions and in parallel. To analyze membrane expressions of P2X7R, we used subcellular Protein Fractionation Kit for Tissues (Thermo Scientific, USA), according to the manufacturer’s instructions. Next, Western blot was performed according to standard procedures.

### Measurement of SNO-thiol- and total thiol on P2X7R and PDI

Modified biotin switch assay was performed with the *S*-nitrosylation Western Blot Kit (ThermoFisher) according to the manufacturer’s protocol. Briefly, lysates were reacted with ascorbate in HENS buffer for specific labeling with iodoTMTzero reagents with MMT pretreatment (for SNO-thiol) or not (for total thiol). Protein labeling can be confirmed by Western blot using TMT antibody. Thereafter, TMT-labeled proteins were purified by Anti-TMT Resin, eluted by TMT elusion buffer, and identified by Western blot according to standard procedures. For technical controls, we omitted ascorbate for each sample. The ratio of SNO-protein or total thiol-protein to total protein was described as *S*-nitrosylation or total thiol levels [7, 8, 22].

### Immunohistochemistry and TUNEL staining

Rats were anesthetized with urethane anesthesia (1.5 g/kg, i.p.) and perfused transcardially with 4% paraformaldehyde in 0.1 M phosphate buffer (PB, pH 7.4). The brains were post-fixed in the same fixative overnight and then cryoprotected and sectioned at 30 μm with a cryostat. Free-floating coronal sections were incubated in PDI antibody in PBS containing 0.3% Triton X-100 overnight at room temperature. Tissue sections were incubated with a mixture of guinea pig anti-NeuN IgG (1:1000, Millipore, #ABN90P)/rabbit anti-extracellular P2X7R IgG (1:200, Alomone labs, APR-008), mouse anti-GFAP IgG (1:1,000, Millipore, #MAB3402)/rabbit anti-extracellular P2X7R IgG, mouse anti-ionizing calcium-binding adaptor molecule 1 (Iba-1, 1:200, Abcam, ab15690)/rabbit anti-extracellular P2X7R IgG, or mouse anti-ionizing calcium-binding adaptor molecule 1 (Iba-1, 1:200, Abcam, ab15690)/rabbit anti-NFκB-S276 (1:100, Abcam, ab106129) antibodies in PBS containing 0.3% Triton X-100 overnight at room temperature. Thereafter, sections were visualized with Cy2- and Cy3-conjugated secondary antibodies. Immunoreaction was observed and analyzed using an AxioImage M2 microscope (Carl Zeiss, Germany). To establish the specificity of the immunostaining, a negative control test was carried out with preimmune serum instead of the primary antibody. TUNEL staining was also performed with the TUNEL apoptosis detection kit (Merck Millipore, USA) according to the manufacturer’s instructions. Following TUNEL reaction, GFAP immunofluorescence staining (mouse anti-GFAP IgG, 1:1,000, Millipore, #MAB3402) was performed. For nuclei counterstaining, we used Vectashield mounting medium with DAPI (Vector, USA). No immunoreactivity was observed for the negative control in any structures. All experimental procedures in this study were performed under the same conditions and in parallel [7, 8, 22].

### Fluoro-Jade B staining

To analyze the neuronal damage, we applied Fluoro-Jade B (FJB) staining. Sections were placed on slides, dried, and immersed in 80% ethanol containing 1% sodium hydroxide. Next, samples were immersed in 70% ethanol solution for 2 min and in purified water for 2 min. After immersion in 0.06% potassium permanganate solution for another 10 min, samples were rinsed with purified water for 2 min. Images were captured using an AxioImage M2 microscope.

### Cell count and measurement of Iba-1/GFAP positive area

The number of cells and Iba-1/GFAP positive area was measured, as previously described [17, 26]. Briefly, sections (10 sections per each animal) were captured, and areas of interest (1 × 10^4^ μm^2^) were selected. Thereafter, cell count and measurement of Iba-1/GFAP-positive area were performed on ×20 magnification images using AxioVision Rel. 4.8 software.

### Statistical analysis

After evaluating the values on normality using Shapiro-Wilk *W* test, comparisons of data among groups were analyzed by Mann–Whitney *U* test (latency of SE onset and behavioral seizure score), Student’s *t* test, repeated-measure ANOVA (total EEG power), or ANOVA followed by Newman–Keuls post hoc test. A *p* < 0.05 is considered to be statistically different.

## Results

### Effect of L-NAME on seizure activity in response to pilocarpine

**Fig. 1:**
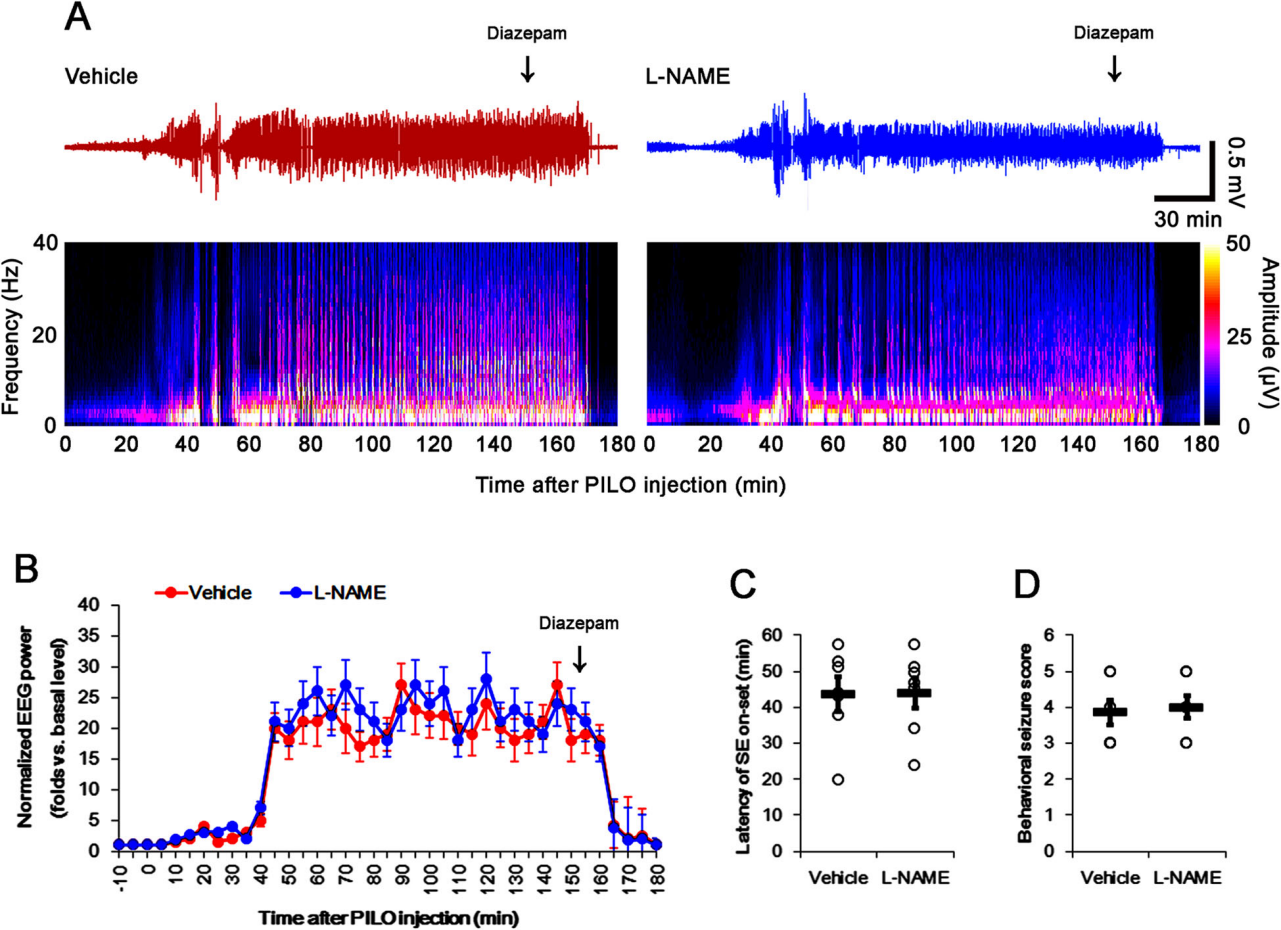
Effect of L-NAME on seizure activity in response to pilocarpine. As compared to vehicle, L-NAME does not affect total EEG power, the latency of SE on-set and behavioral seizure severity (seizure score) in response to pilocarpine. (**a**) Representative EEG (upper panels) and frequency-power spectral temporal maps (lower panels) in response to pilocarpine (n = 7, respectively). (**b**-**d**) Quantification of total EEG power (B), the latency of SE on-set (**c**) and behavioral seizure severity (**d**) in response to pilocarpine (n = 7, respectively). Open circles indicate each individual value. Horizontal bars indicate mean value. Error bars indicate S.E.M

First, we evaluated the effect of L-NAME pretreatment on seizure activity in response to pilocarpine. In vehicle-treated animals, the latency of SE onset was 43.7 min (Fig. 1). After SE onset, behavioral seizure severity was Racine’s score 3.9 (Fig. 1). L-NAME pretreatment did not influence total EEG power, the latency of SE onset, and the behavioral seizure score (Fig. 1).

### L-NAME inhibits *S*-nitrosylation of P2X7R and PDI under physiological and post-SE conditions

**Fig. 2:**
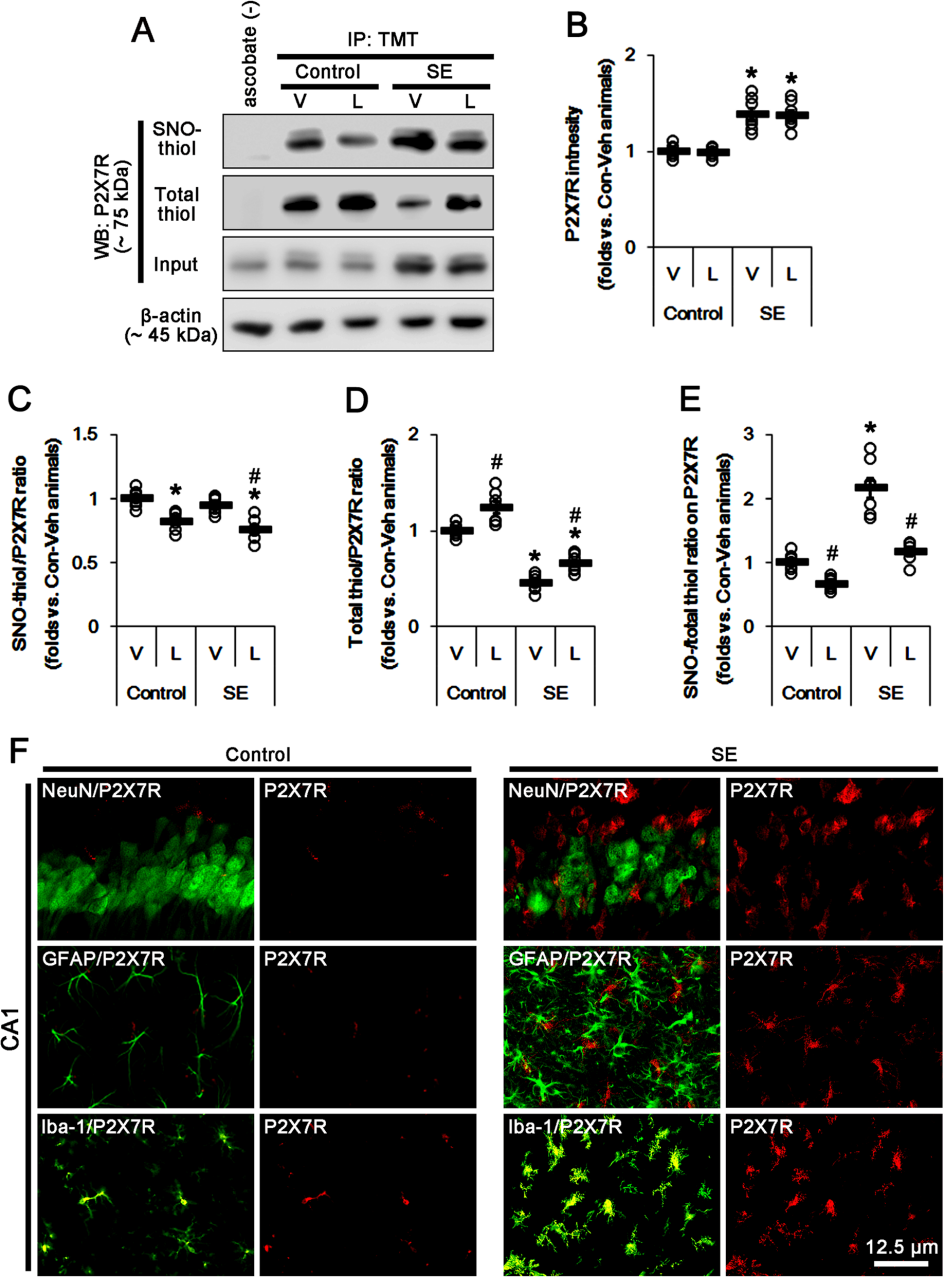
The effects of L-NAME on the amounts of SNO-thiol- and total thiol-P2X7R following SE. As compared to vehicle (V), L-NAME (L) reduces the amount of SNO-thiol-P2X7R and increased that of total thiol-P2X7R without altering P2X7R expression under physiological condition. Thus, *S*-nitrosylation ratio of P2X7R is decreased. SE upregulates P2X7R expression and *S*-nitrosylation ratio of P2X7R due to the reduced the amount of total thiol without the altered that of SNO-thiol-P2X7R. L-NAME inhibits these alterations induced by SE, except P2X7R expression. **a** Representative Western blot for *S*-nitrosylation and thiolization on P2X7R and its expression. **b-e** Quantification of analyses of *S*-nitrosylation and thiolization on P2X7R and its expression. Open circles indicate each individual value. Horizontal bars indicate the mean value. Error bars indicate SEM (*^#^*p* < 0.05 vs. control animals and vehicle, respectively; *n* = 7, respectively). **f** Representative photos of the localization of P2X7R in the CA1 region following SE. Upregulation of P2X7R expression induced by SE is restricted to microglia

Next, we investigated the effects of L-NAME on the *S*-nitrosylated (SNO-) thiol and the total (-SNO + -SH) thiol on P2X7R under physiological condition. L-NAME diminished the amount of SNO-thiol on P2X7R (SNO-thiol-P2X7R) without altering P2X7R expression level (*t*_(12)_ = 5.04, *p* = 0.0002 vs. vehicle, Student’s *t* test, *n* = 7, respectively; Fig. 2a–c and Supplementary figure S1). However, L-NAME increased the amount of total thiol-P2X7R (total thiol-P2X7R, *t*_(12)_ = 3.65, *p* = 0.003 vs. vehicle, Student’s *t* test, *n* = 7, respectively; Figs. 2a and 1d and Supplementary figure S1); thus, it decreased the *S*-nitrosylation ratio (SNO-thiol/total thiol) on P2X7R (*t*_(12)_ = 5.46, *p* = 0.0001 vs. vehicle, Student’s *t* test, *n* = 7, respectively; Fig. 2a and e and Supplementary figure S1). SE increased P2X7R expression (*t*_(12)_ = 5.51, *p* = 0.0001 vs. control, Student’s *t* test, *n* = 7, respectively; Fig. 2a, b and Supplementary figure S1). Consistent with our previous study [27], immunohistochemical studies revealed that the upregulation of P2X7R was mainly observed in microglia, but not in neurons or astrocytes (Fig. 2f).

Although SE elevated SNO-thiol level, it did not affect the amount of SNO-thiol-P2X7R due to the increased P2X7R expression (Fig. **2**a, c). Since SE reduced the amount of total thiol-P2X7R (*t*_(12)_ = 13.57, *p* < 0.0001 vs. control, Student’s *t* test, *n* = 7, respectively; Fig. **2**a, d and Supplementary figure S1), the *S*-nitrosylation ratio of P2X7R was significantly increased following SE (*t*_(12)_ = 7.02, *p* < 0.0001 vs. control, Student’s *t* test, *n* = 7, respectively; Fig. **2**a, e and Supplementary figure S1). Similar to the cases under physiological condition, L-NAME reduced the amount of SNO-thiol-P2X7R (*t*_(12)_ = 4.74, *p* = 0.0004 vs. vehicle, Student’s *t* test, *n* = 7, respectively; Fig. **2**a, c and Supplementary figure S1) and elevated the amount of total thiol-P2X7R without affecting P2X7R expression following SE (*t*_(12)_ = 4.44, *p* = 0.0008 vs. vehicle, Student’s *t* test, *n* = 7, respectively; Fig. **2**a, d and Supplementary figure S1). Thus, L-NAME abrogated the increase in *S*-nitrosylation ratio of P2X7R induced by SE (*t*_(12)_ = 6.01, *p* < 0.0001 vs. vehicle, Student’s *t* test, *n* = 7, respectively; Fig. **2**a, e and Supplementary figure S1). These findings indicate that L-NAME may decrease the *S*-nitrosylation ratio of P2X7R by elevating the amount of total thiol-P2X7R as well as diminishing the amount of SNO-thiol-P2X7R. Taken together, our findings also suggest that L-NAME may increase the amount of free thiol-P2X7R (in other words, the reduction of disulfide bonds on P2X7R) under physiological and post-SE conditions.

### *S*-nitrosylation of P2X7R is involved in microglial activation and astroglial loss following SE

**Fig. 3:**
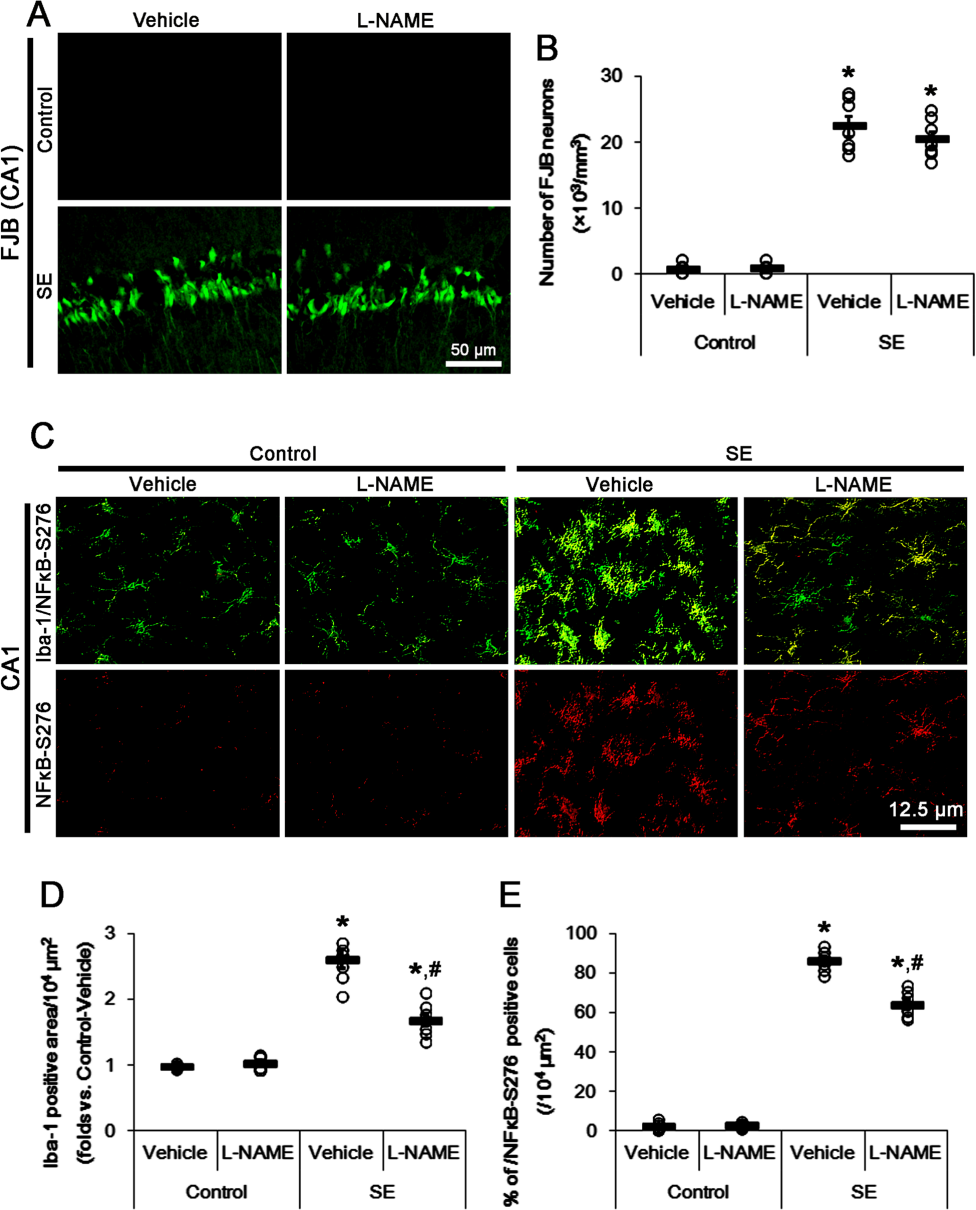
The effects of L-NAME on neuronal death and microglial activation following SE. L-NAME attenuates microglial activation by inhibiting p65-S276 NF-κB phosphorylation, while it does not affect CA1 neuronal death, following SE. **a** Representative photos for FJB-positive degenerating CA1 neurons induced by SE. **b** Quantification of analyses of the effect of L-NAME on SE-induced CA1 neurons. Open circles indicate each individual value. Horizontal bars indicate the mean value. Error bars indicate SEM (*n* = 7, respectively). **c** Representative photos for microglial p65-S276 NF-κB phosphorylation in the CA1 region following SE. **d**, **e** Quantification of analyses of the effect of L-NAME on microglial activation (**d**) and p65-S276 NF-κB phosphorylation in microglia (**e**). Open circles indicate each individual value. Horizontal bars indicate the mean value. Error bars indicate SEM (*^#^*p* < 0.05 vs. control animals and vehicle, respectively; *n* = 7, respectively)

Since P2X7R is involved in the neuronal, microglial, and astroglial responses to SE [12–16], we explored whether the blockade of *S*-nitrosylation of P2X7R by L-NAME affects these post-SE events. In the present study, SE led to massive CA1 neuronal death (*F*_(3,24)_ = 155.21, *p* < 0.0001 vs. control, one-way ANOVA, *n* = 7, respectively; Fig. 3a, b). As compared to vehicle, L-NAME did not affect CA1 neuronal death induced by SE (Fig. 2a, b). Following SE, microglia appeared hypertrophic and amoeboid shapes indicating the phagocytic and inflammatory/cytotoxic activities [28] (*F*_(3,24)_ = 75.43, *p* < 0.0001 vs. control, one-way ANOVA, *n* = 7, respectively; Fig. 3c, d). In L-NAME-treated animals, microglia showed hyper-ramified shapes with shrunken cell bodies and slender processes indicating the intermediate between the resting and reactive states [28] (*F*_(1,12)_ = 29.63, *p* = 0.0002 vs. vehicle, one-way ANOVA, *n* = 7, respectively; Fig. 3c, d). These findings indicate that L-NAME may effectively attenuate SE-induced microglial activation.

Because p65-serine (S) 276 NF-κB phosphorylation enhances its transactivation potential and interaction with cAMP response element-binding (CREB)-binding protein, which is important for the microglial activation [29, 30], we also investigated the effects of L-NAME on p65-S276 NF-κB phosphorylation in microglia. SE increased p65-S276 NF-κB phosphorylation in activated microglia (*F*_(3,24)_ = 685.15, *p* < 0.0001 vs. control, one-way ANOVA, *n* = 7, respectively; Fig. 3c, e), which was attenuated by L-NAME (*F*_(1,12)_ = 49.3, *p* < 0.0001 vs. vehicle, one-way ANOVA, *n* = 7, respectively; Fig. 3c, e). These findings indicate that the reducing *S*-nitrosylation ratio of P2X7R by L-NAME may inhibit SE-induced microglial activation.

**Fig. 4:**
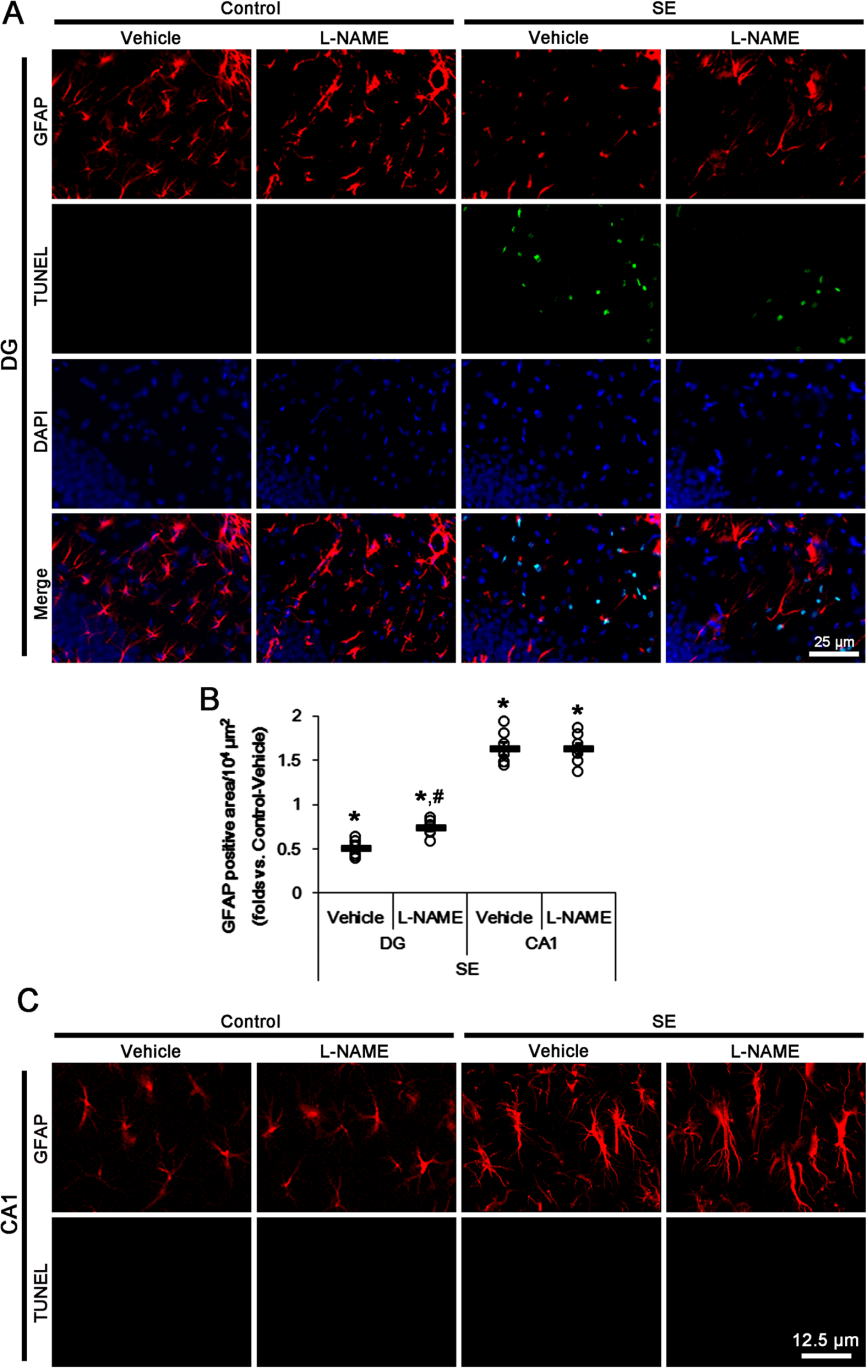
The effects of L-NAME on astroglial death and reactive astrogliosis in the hippocampus following SE. SE leads to astroglial apoptosis in the molecular layer of the dentate gyrus and reactive astrogliosis in the CA1 region, respectively. L-NAME ameliorates SE-induced astroglial apoptosis, but not reactive astrogliosis. **a** Representative photos for TUNEL-positive astrocytes in the dentate gyrus following SE. **b** Quantification of analyses of the effect of L-NAME on GFAP-positive area following SE. Open circles indicate each individual value. Horizontal bars indicate the mean value. Error bars indicate SEM (*^#^*p* < 0.05 vs. control animals and vehicle, respectively; *n* = 7, respectively). **c** Representative photos for double immunostaining for TUNEL and GFAP in the CA1 region following SE. Reactive CA1 astrocytes do not show TUNEL signals following SE

On the other hand, SE results in a massive degeneration of astrocytes in the molecular layer of the dentate gyrus (DG astrocytes), but reactive astrogliosis in the stratum radiatum of the CA1 regions (CA1 astrocytes). Since blockade of P2X7R functionality attenuates DG astroglial degeneration induced by SE [14], we also evaluated the effects of L-NAME on SE-induced DG astroglial apoptosis. Following SE, the GFAP-positive area in the molecular layer of the dentate gyrus was reduced to ~ 50% of that in non-SE animals, accompanied by the increase in the number of TUNEL-positive astrocytes (*F*_(3,24)_ = 63.84, *p* < 0.0001 vs. control, one-way ANOVA, *n* = 7, respectively; Fig. **4**a, b). L-NAME diminished GFAP-deleted area in the molecular layer of the dentate gyrus and the number of TUNEL-positive astrocytes (*F*_(1,12)_ = 24.2, *p* = 0.0004 vs. vehicle, one-way ANOVA, *n* = 7, respectively; Fig. **4**a, b). These findings demonstrate that L-NAME may attenuate DG astroglial apoptosis induced by SE. In contrast to DG astrocytes, CA1 astrocytes showed typical reactive astrogliosis following SE, which astrocytes had the unevenly thick processes with ragged edges(*F*_(3,24)_ = 44.99, *p* < 0.0001 vs. control, one-way ANOVA, *n* = 7, respectively; Fig. **4**b, c), which was unaffected by L-NAME (Fig. **4**b, c). These findings are consistent with a previous study [31] demonstrating that L-NAME influences the induction of reactive gliosis in microglia, but not astrocytes. Therefore, our findings suggest that *S*-nitrosylation may reinforce P2X7R-mediated microglial activation as well as DG astroglial apoptosis following SE.

### L-NAME decreases *S*-nitrosylation of PDI and PDI-P2X7R bindings under physiological and post-SE conditions

**Fig. 5:**
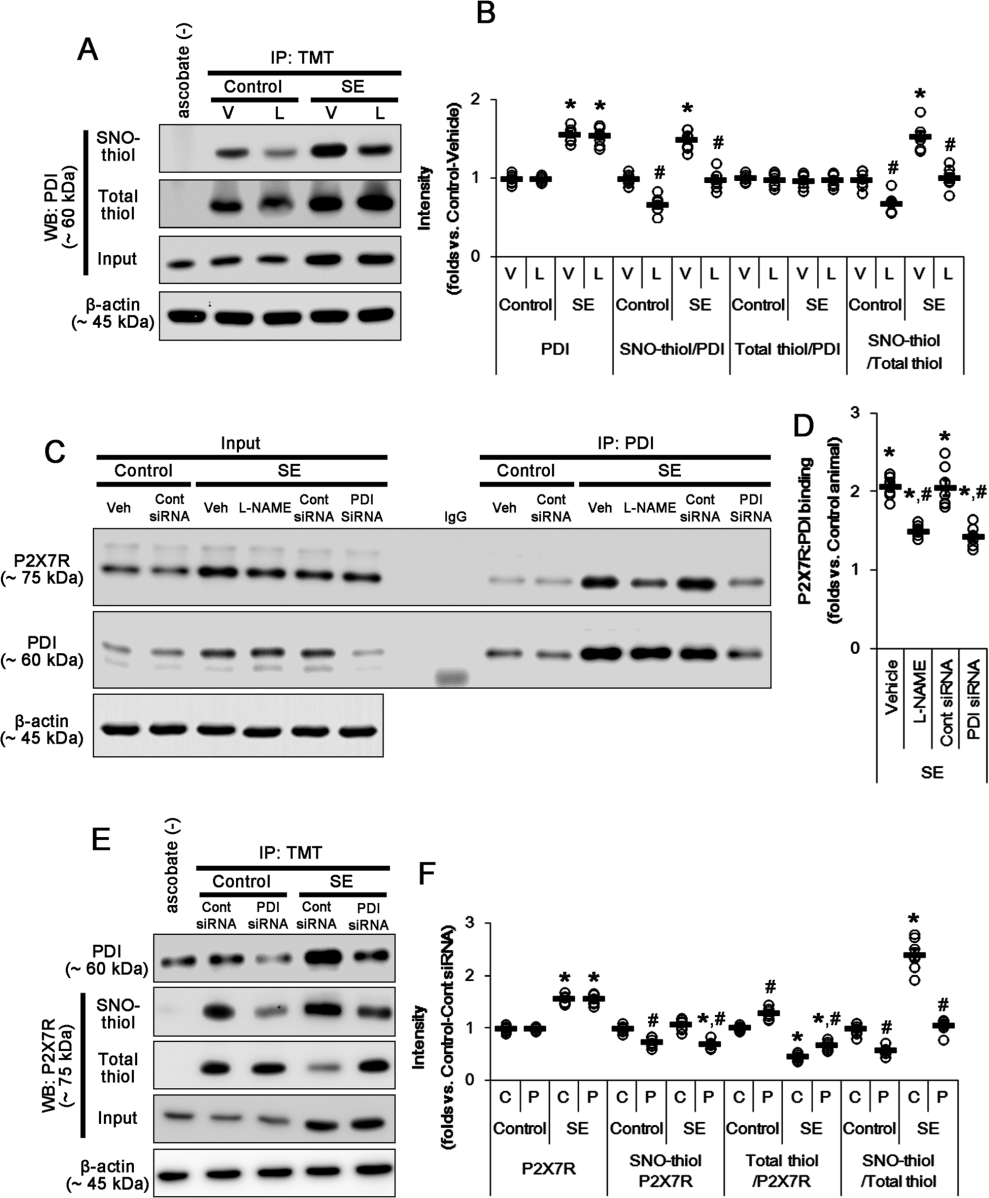
The effects of L-NAME and/or PDI knockdown on the amounts of SNO-thiol and total thiol-PDI or -P2X7R, and PDI-P2X7R bindings following SE. As compared to vehicle (V), L-NAME (L) reduces the amount of SNO-thiol-PDI and PDI-P2X7R bindings without affecting PDI expression and the amount of total thiol-PDI under physiological condition. Thus, *S*-nitrosylation ratio of PDI is decreased. L-NAME abrogates SE-induced alterations in PDI-P2X7R bindings and *S*-nitrosylation ratio of PDI, except PDI expression. As compared to control siRNA (C), PDI siRNA (P) abolishes the changes in PDI-P2X7R bindings and *S*-nitrosylation ratio of P2X7R following SE, except its expression. **a** Representative Western blot for *S*-nitrosylation and thiolization on PDI and its expression. **b** Quantification of analyses of *S*-nitrosylation and thiolization on PDI and its expression. Open circles indicate each individual value. Horizontal bars indicate the mean value. Error bars indicate SEM (*^#^*p* < 0.05 vs. control animals and vehicle, respectively; *p < 0.05 vs. vehicle; *n* = 7, respectively). **c** Representative Western blot for the PDI-P2X7R bindings following SE. **d** Quantification of analyses of PDI-P2X7R bindings. Open circles indicate each individual value. Horizontal bars indicate the mean value. Error bars indicate SEM (*^#^*p* < 0.05 vs. control animals and vehicle or control siRNA, respectively; *n* = 7, respectively). **e** Representative Western blot for *S*-nitrosylation and thiolization on P2X7R and its expression. **f** Quantification of analyses of *S*-nitrosylation and thiolization on P2X7R and its expression. Open circles indicate each individual value. Horizontal bars indicate the mean value. Error bars indicate SEM (*^#^*p* < 0.05 vs. control animals and control siRNA, respectively; *n* = 7, respectively)

Since NO does not form a SNO via a direct reaction with -SH group [31] and SNO-PDI acts as a NO donor [8, 25], we investigated whether PDI modulates *S*-nitrosylation of P2X7R under physiological and post-SE conditions. Similar to the case of P2X7R, L-NAME diminished the amount of SNO-thiol-PDI (*t*_(12)_ = 6.9, *p* < 0.0001 vs. vehicle, Student’s *t* test, *n* = 7, respectively) and the *S*-nitrosylation ratio of PDI without changing PDI expression under physiological condition (*t*_(12)_ = 4.94, *p* = 0.0003 vs. vehicle, Student’s *t* test, *n* = 7, respectively; Fig. **5**a, b and Supplementary figure S2). Consistent with previous studies [7, 22, 32], SE increased PDI expressions (*t*_(12)_ = 12.77, *p* < 0.0001 vs. control, Student’s *t* test, *n* = 7, respectively), the amount of SNO-thiol-PDI (*t*_(12)_ = 9.3, *p* < 0.0001 vs. control, Student’s *t* test, *n* = 7, respectively) and the *S*-nitrosylation ratio of PDI in total hippocampal tissue without altering the amount of total thiol-PDI (*t*_(12)_ = 7.42, *p* < 0.0001 vs. control, Student’s *t* test, *n* = 7, respectively; Fig. **5**a, b and Supplementary figure S2). SE also elevated the PDI-P2X7R bindings (*t*_(12)_ = 18.14, *p* < 0.0001 vs. control, Student’s *t* test, *n* = 7, respectively; Fig. **5**c, d and Supplementary figure S3). Similar to the cases under physiological condition, L-NAME reduced the amount of SNO-thiol-PDI (*t*_(12)_ = 7.83, *p* < 0.0001 vs. vehicle, Student’s *t* test, *n* = 7, respectively) and the *S*-nitrosylation ratio of PDI following SE (*t*_(12)_ = 6.57, *p* < 0.0001 vs. vehicle, Student’s *t* test, *n* = 7, respectively; Fig. **5**a, b and Supplementary figure S2). L-NAME did not affect the amount of total thiol-PDI and SE-induced upregulation of PDI expression (Fig. **5**a, b). However, L-NAME abrogated the increased PDI-P2X7R bindings induced by SE (*t*_(12)_ = 9.4, *p* < 0.0001 vs. vehicle, Student’s *t* test, *n* = 7, respectively; Fig. **5**c, d and Supplementary figure S3). These findings indicate that the amount of SNO-thiol-P2X7R may closely relevant to the *S*-nitrosylation ratio of PDI and that SNO-PDI may be one of the NO donors for P2X7R.

### PDI knockdown inhibits reduction and *S*-nitrosylation of P2X7R under physiological and post-SE conditions

**Fig. 6:**
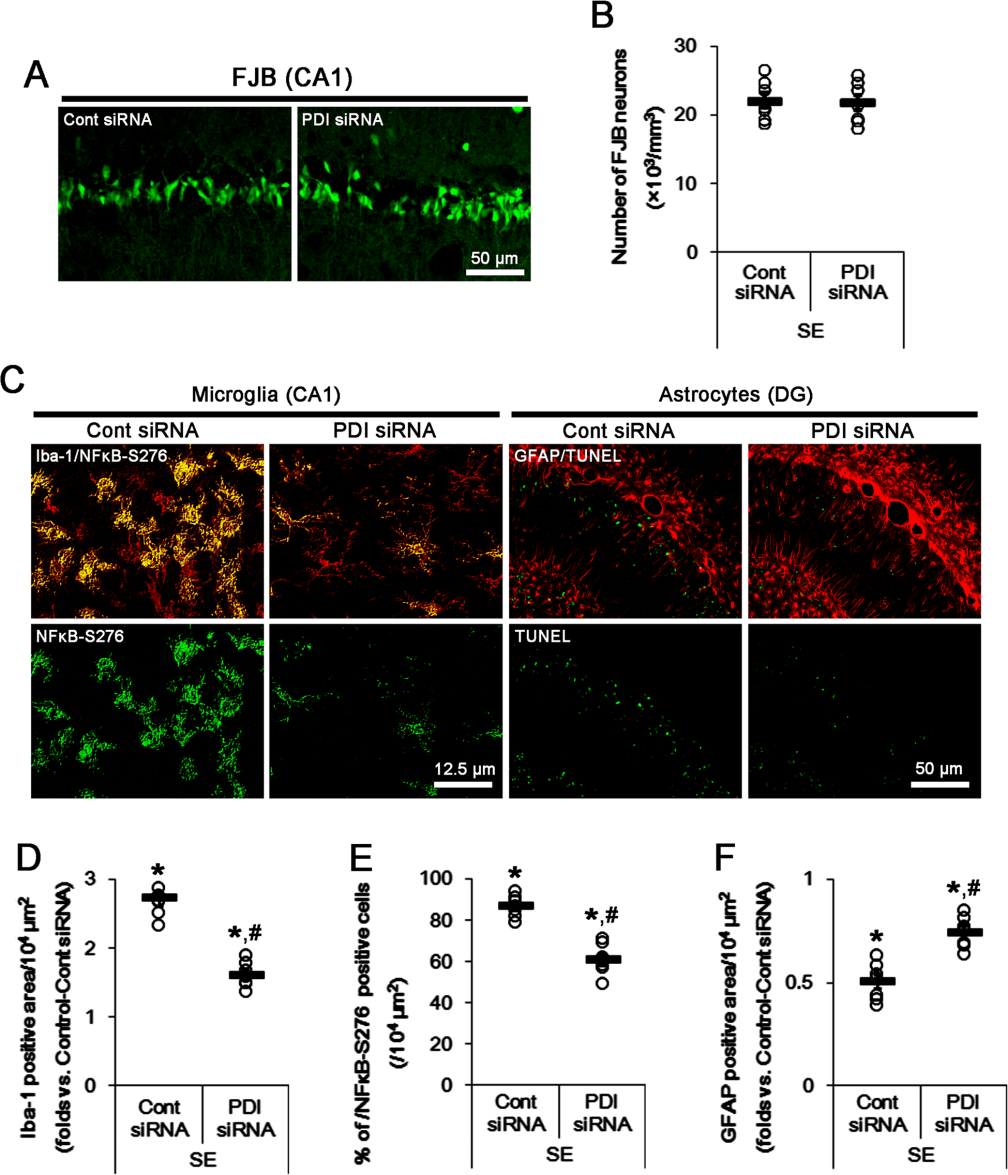
The effects of PDI knockdown on neuronal death, microglial activation, and astroglial apoptosis following SE. PDI siRNA inhibits microglial activation by inhibiting p65-S276 NF-κB phosphorylation and attenuates astroglial apoptosis in the dentate gyrus following SE, while it does not affect SE-induced neuronal death. **a** Representative photos for FJB-positive degenerating CA1 neurons following SE. **b** Quantification of analyses of the effect of PDI siRNA on SE-induced CA1 neuronal degeneration. Open circles indicate each individual value. Horizontal bars indicate the mean value. Error bars indicate SEM (*n* = 7, respectively). **c** Representative photos for p65-S276 NF-κB phosphorylation in microglia in the CA1 region (left panels) and TUNEL-positive astrocytes in the dentate gyrus (right panels) following SE. **d-f** Quantification of analyses of the effect of PDI siRNA on microglial activation (**d**), p65-S276 NF-κB phosphorylation in microglia (**e**), and astroglial apoptosis in the dentate gyrus (**f**). Open circles indicate each individual value. Horizontal bars indicate the mean value. Error bars indicate SEM (*^#^*p* < 0.05 vs. control animals and control siRNA, respectively; *n* = 7, respectively)

To validate directly the role of PDI in *S*-nitrosylation of P2X7R, we applied PDI knockdown to normal animals. PDI knockdown reduced PDI expression level to ~ 42% of control siRNA level under physiological condition without altering P2X7R protein level (*t*_(12)_ = 14.57, *p* < 0.0001 vs. control siRNA, Student’s *t* test, *n* = 7, respectively; Fig. **4**e and Supplementary figure S2). PDI siRNA reduced the amount of SNO-thiol-P2X7R (*t*_(12)_ = 6.14, *p* < 0.0001 vs. control siRNA, Student’s *t* test, *n* = 7, respectively), but increased the amount of total thiol-P2X7R (*t*_(12)_ = 6.41, *p* < 0.0001 vs. control siRNA, Student’s *t* test, *n* = 7, respectively). Thus, PDI siRNA decreased *S*-nitrosylation ratio of P2X7R (*t*_(12)_ = 7.95, *p* < 0.0001 vs. control siRNA, Student’s *t* test, *n* = 7, respectively; Fig. **5**e, f and Supplementary figure S2). PDI siRNA restored the upregulated PDI expression level induced by SE to basal level without changing P2X7R protein level (*t*_(12)_ = 13.28, *p* < 0.0001 vs. control siRNA, Student’s *t* test, *n* = 7, respectively; Fig. **5**e, f and Supplementary figure S2). PDI knockdown also ameliorated the increased PDI-P2X7R bindings induced by SE (*t*_(12)_ = 5.57, *p* = 0.0001 vs. control siRNA, Student’s *t* test, *n* = 7, respectively; Fig. **5**c, d, Supplementary figure S2 and S3). It also declined the amounts of SNO-thiol-P2X7R (*t*_(12)_ = 7.61, *p* = 0.0001 vs. control siRNA, Student’s *t* test, *n* = 7, respectively) and *S*-nitrosylation ratio of P2X7R (*t*_(12)_ = 10.72, *p* = 0.0001 vs. control siRNA, Student’s *t* test, *n* = 7, respectively), but increased total thiol-P2X7R following SE (*t*_(12)_ = 5.57, *p* = 0.0001 vs. control siRNA, Student’s *t* test, *n* = 7, respectively; Fig. **5**e, f and Supplementary figure S2). Furthermore, PDI knockdown attenuated microglial activation (*t*_(12)_ = 8.9, *p* < 0.0001 vs. control siRNA, Student’s *t* test, *n* = 7, respectively), p65-S276 NF-κB phosphorylation in microglia (*t*_(12)_ = 7.49, *p* < 0.0001 vs. control siRNA, Student’s *t* test, *n* = 7, respectively), and DG astroglial apoptosis (*t*_(12)_ = 5.44, *p* = 0.0002 vs. control siRNA, Student’s *t* test, *n* = 7, respectively) induced by SE, while it did not prevent SE-induced CA1 neuronal degeneration (Fig. **6**a–f). Because PDI knockdown does not affect NO synthesis [8], these findings indicate that PDI knockdown may directly reduce *S*-nitrosylation of P2X7R. Therefore, our findings suggest that PDI may play a role as a NO transporter to P2X7R and that *S*-nitrosylation of P2X7R may positively modulate reactive microgliosis and DG astroglial loss following SE.

### *S*-nitrosylation of P2X7R facilitates its trafficking following SE

**Fig. 7:**
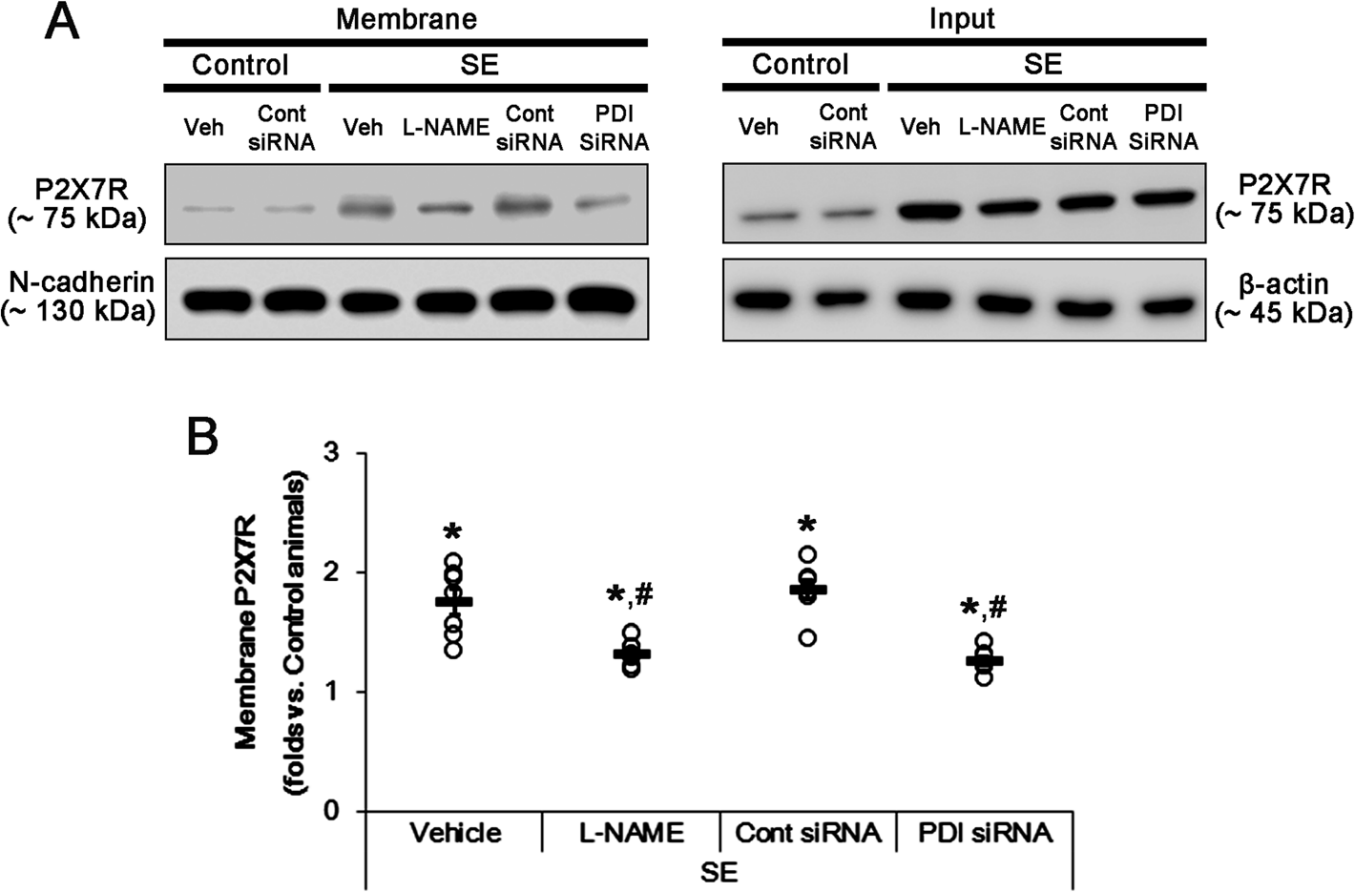
The effects of L-NAME and PDI knockdown on the surface expression of P2X7R. As compared to control animals, SE increases the membrane P2X7R expressions, which are abrogated by both L-NAME and PDI siRNA. **a** Representative Western blot for surface expression of P2X7R following SE. **b** Quantification of analyses of P2X7R expression in membrane fractions. Open circles indicate each individual value. Horizontal bars indicate the mean value. Error bars indicate SEM (*^#^*p* < 0.05 vs. control animals and vehicle or control siRNA, respectively; *n* = 7, respectively)

The remaining question is how *S*-nitrosylation of P2X7R regulates its functionality. Since disulfide bond formations of P2X7R are needed for the trafficking to the cell surface [20], we explored the effects of L-NAME and PDI siRNA on the cell surface expression of P2X7R. In the present study, SE elevated cell surface expression of P2X7R in vehicle- and control siRNA-infused animals (*F*_(3,24)_ = 44.78, *p* < 0.0001 vs. control, one-way ANOVA, *n* = 7, respectively; Fig. 7a, b and Supplementary figure S4). Both L-NAME and PDI siRNA ameliorated the membrane P2X7R expression induced by SE (*F*_(3,24)_ = 17.03, *p* < 0.0001 vs. vehicle or control siRNA, respectively, one-way ANOVA, *n* = 7, respectively; Fig. 7a, b and Supplementary figure S4). Thus, these findings indicate that PDI-mediated *S*-nitrosylation may facilitate the trafficking of P2X7R and upregulate its surface expression.

## Discussion

**Fig. 8:**
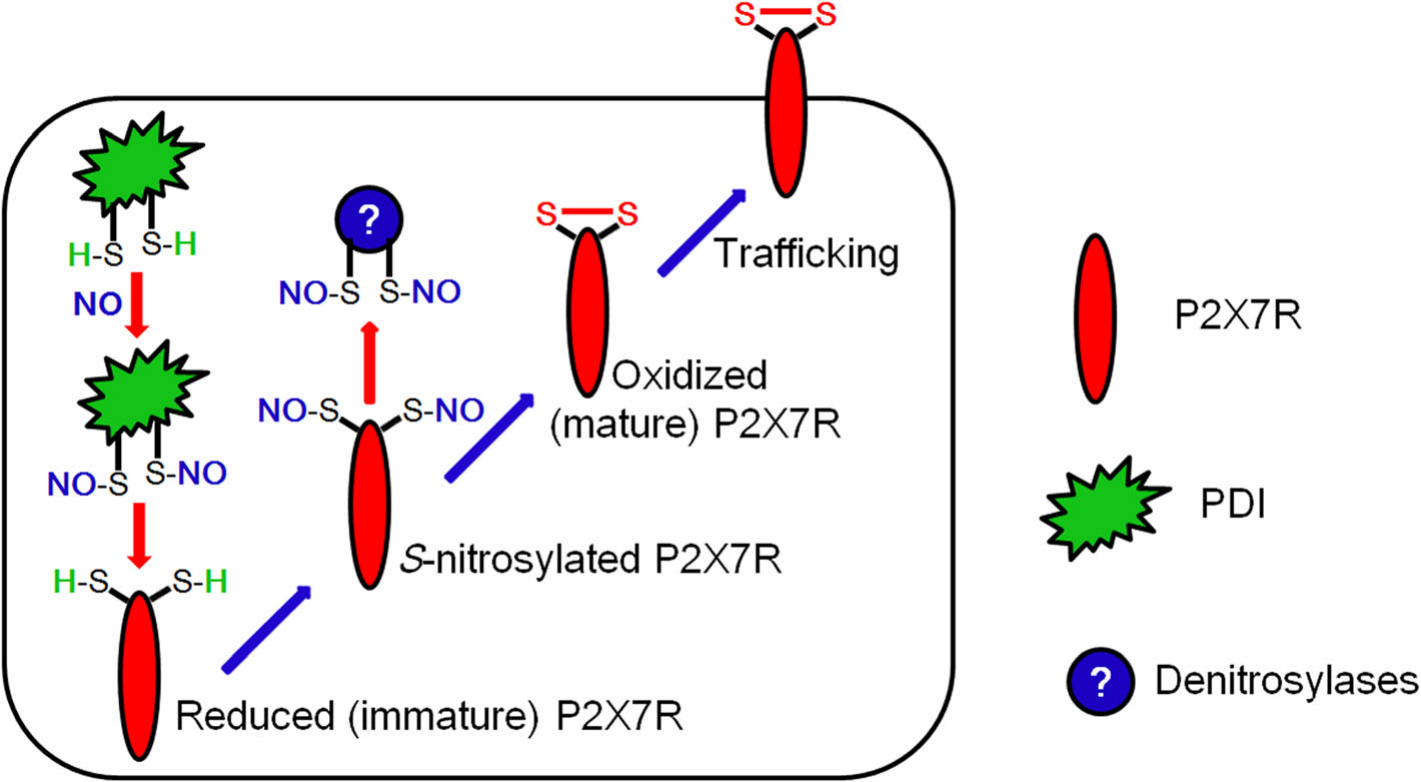
Scheme of the role of PDI-mediated *S*-nitrosylation of P2X7R in its trafficking to the cell membrane. SE elevates NO synthesis, which *S*-nitrosylates PDI. SNO-PDI transfers NO to the reduced (immature) P2X7R. In turn, SNO-P2X7R is oxidized by denitrosylases (presumably Trx-1 [8]), which facilitates its trafficking to the cell surface

The major findings in the present study are that PDI nitrosylated reduced (immature) P2X7R under physiological and post-SE conditions. *S*-nitrosylation facilitated disulfide bonds formations of P2X7R. Subsequently, oxidized (mature) P2X7R increased its trafficking on the cell membrane, which exerted microglial activation and DG astroglial loss following SE (Fig. 8).

P2X7R plays a critical role in both physiological responses to tissue damage and inflammation and in the pathogenesis and progression of a diversity of associated conditions [9–11]. The disulfide bonds in extracellular domain of P2X7R are required for its trafficking to the cell surface and its action as a scavenger receptor [19–21]. Thus, post-translational modifications of disulfide bonds would affect P2X7R-mediated events, which are much less defined. In our previous studies [7, 8, 27], SE results in the prolonged NO synthesis and the sustained P2X7R activation. Since the inhibitions of NO synthesis and P2X7R activity induced by SE attenuate microglial activation [14, 17, 27, 33, 34], it is likely that these post-SE events may act in concert to regulate microglia-mediated neuroinflammation. In the present study, we found that P2X7R was nitrosylated, which was abolished by L-NAME and PDI siRNA, under physiological and post-SE conditions. *S*-nitrosylation results in conformational changes in protein structure, which influence additional post-translational modifications of cysteine residues. In particular, *S*-nitrosylation facilitates disulfide bond formation (oxidation) between cysteine residues [35–37]. Regarding that disulfide bond formations are required for the trafficking of P2X7R [20], it is likely that SE-induced prolonged NO production may modulate microglial activation by elevating P2X7R surface expression. Indeed, the present study reveals that the decrease in the amounts of SNO-thiols on P2X7R by L-NAME and PDI siRNA ameliorated SE-induced microglial transformation and p65-S276 NF-κB phosphorylation, which are indicative of microglial activation [28–30]. Therefore, these findings suggest that prolonged NO synthesis may nitrosylate P2X7R and upregulate its surface expression in microglia, which may exert microglia-mediated inflammatory responses to SE.

The present study also demonstrates that both L-NAME and PDI siRNA mitigated SE-induced DG astroglial loss, accompanied by the reduced surface expression of P2X7R. Although immunohistochemical studies have reported microglial-restricted localization of P2X7R in the brain [27, 38], pharmacological and physiological data reveal that P2X7R is also expressed in astrocytes and neurons [12–15, 39–41]. Indeed, P2X7R antagonists attenuate DG astroglial degeneration induced by SE [14]. Thus, our findings suggest that *S*-nitrosylation of P2X7R may be involved in SE-induced DG astroglial apoptosis. However, it cannot be excluded the possibility that PDI knockdown would directly ameliorate DG astroglial apoptosis induced by SE. This is because PDI is involved in the release of apoptosis-inducing factor (AIF) from the mitochondria [42] that triggers the caspase-independent astroglial apoptosis [32]. Further studies are needed to elucidate the role of PDI itself in SE-induced astroglial apoptosis.

On the other hand, *S*-nitrosylation also inhibits palmitoylation (a post-translational modification of cysteine by binding to a 16-carbon fatty acid palmitate) of thiols under severe nitrosative conditions [1, 43, 44]. Thus, it is plausible that *S*-nitrosylation would also inhibit palmitoylation of P2X7R. If true, *S*-nitrosylation would decrease the amounts of P2X7R in membrane fraction and its total expression following SE. This is because palmitoylation at C-terminus increases the trafficking of P2X7R to the cell membrane and non-palmitoylated P2X7R is rapidly degraded [45]. In the present study, however, SE elevated cell surface expression of P2X7R concomitant with the increases in *S*-nitrosylation and disulfide bond formation, which were ameliorated by L-NAME and PDI knockdown. Thus, our findings suggest that PDI-mediated *S*-nitrosylation may facilitate the trafficking of P2X7R and upregulate its surface expression independent of palmitoylation.

SE increases SNO-PDI level without changing thioredoxin-1 (Trx-1, a denitrosylating enzyme) activity [8]. *S*-nitrosylation of PDI inhibits its activity that controls the redox state of protein thiols or reactive disulfides [43, 46]. Regardless of its redox activity, SNO-PDI transfers NO onto SH groups of various proteins [8, 25]. In the present study, L-NAME diminished the amount of SNO-thiol-PDI without affecting that of total thiol-PDI. Furthermore, L-NAME reduced the amount of SNO-thiol-P2X7R and elevated the amount of total thiol-P2X7R (indicating the reduction of disulfide bonds on P2X7R). Thus, these findings indicate L-NAME may also decrease the surface expression of P2X7R by enhancing the reducing activity of PDI. Since PDI inhibits *N*-methyl-d-aspartate receptor (NMDAR) activity via sulfhydration (reduction) of disulfide bonds [7, 22], it is plausible that the L-NAME would also attenuate microglial activation and DG astroglial apoptosis by facilitating PDI-mediated reduction of surface P2X7R. However, P2X7R-mediated currents are unaffected by the reduction of disulfide bonds on surface P2X7R [47]. Therefore, our findings suggest that PDI-mediated *S*-nitrosylation of P2X7R may reinforce SE-induced microglial activation and DG astroglial loss by increasing its surface expression, independent of activities of PDI and surface P2X7R.

Consistent with a previous study [8], the present study shows that L-NAME did not influence SE-induced CA1 neuronal death. Unlike other cell populations, SE downregulates PDI expression and SNO-PDI level in CA1 neurons, which result in the impaired dynamin-related protein 1 (DRP1)-mediated mitochondrial dynamics [8, 32]. Furthermore, SE-induced CA1 neuronal death is unaffected by activities of P2X7R and NOS [5, 48]. With respect to these reports, it is likely that PDI-mediated P2X7R regulation may not be directly involved in SE-induced CA1 neuronal degenerations.

On the other hand, P2X7R contributes to pyroptosis that is a type of cell death mediated by inflammatory caspases, such as human and mouse caspase-1, human caspase-4/5, or mouse caspase-11 [49, 50]. In the canonical pathway, caspase-1 activation is mediated by NOD-, LRR-, and pyrin domain-containing protein 3 (NLRP3) [49, 50]. Considering that degenerating neurons release ATP, it is hypothesized that P2X7R/NLRP3/caspase-1 axis may mediate pyroptosis: Released ATP from injured neurons (or other cells) would lead to the K^+^ efflux (one of NLRP3 activators) by P2X7R activation and induce NLRP3 activation with the subsequent formation of active caspase-1, which would cleave the pro-domain of inflammatory cytokines and the N-terminal domain of gasdermin D (GSDMD-NT) from gasdermin D (GSDMD, the pyroptosis executioner). Cleaved GSDMD-NT would translocate to the inner leaflet of the plasma membrane and bind to phosphatidylinositol phosphates and/or phosphatidylserine, which would lead to GSDMD-NT oligomerization. In turn, oligomerized GSDMD-NT would trigger the large transmembrane pore formation that would be responsible for membrane permeabilization during pyroptosis [51, 52]. Therefore, it is plausible that PDI-mediated P2X7R regulation may modulate pyroptosis through NLRP3 inflammasome activation following SE. The specific roles of P2X7R in NLRP3 inflammasome function and pyroptosis in the distinct brain cell populations (neurons, microglia, astrocytes, and oligodendroglia) following SE would be investigated in future studies.

## Conclusions

In conclusion, we demonstrate that PDI-mediated *S*-nitrosylation affected the P2X7R activity: The amount of SNO-thiol-P2X7R was reduced by L-NAME and PDI siRNA. These modes of regulations abrogated the disulfide bond formation and the surface expression of P2X7R. In addition, *S*-nitrosylation and oxidation of P2X7R promoted microglial activation and induced DG astroglial apoptosis following SE. Therefore, we suggest that the targeting of P2X7R to *S*-nitrosylation and/or redox status may be one of the important therapeutic strategies of neuroinflammation and astroglial death in disease states.

## Supplementary Information

**Additional file 1.**

## Data Availability

The authors agree to share all data generated from this study.

## Abbreviations

AIF: Apoptosis-inducing factor
CREB: cAMP response element-binding
DRP1: Dynamin-related protein 1
ER: Endoplasmic reticulum
FJB: Fluoro-Jade B
IL-1β: Interleukin-1β
NLRP3: NOD-, LRR-, and pyrin domain-containing protein 3
NMDAR: *N*-methyl-d-aspartate receptor
NO: Nitric oxide
NF-κB: Nuclear factor-κB
L-NAME: *N*^ω^-nitro-l-arginine methyl ester hydrochloride
P2X7R: P2X7 receptor
PDI: Protein disulfide isomerase
RIPA: Radioimmune precipitation buffer
-SNO: *S*-nitrosothiols
SD: Sprague-Dawley
SE: Status epilepticus
-SH: Sulfhydryl
Trx-1: Thioredoxin-1

## References

[1] Hess DT, Matsumoto A, Kim SO, Marshall HE, Stamler JS (2005). Protein S-nitrosylation: purview and parameters. Nat Rev Mol Cell Biol..

[2] Nakamura T, Tu S, Akhtar MW, Sunico CR, Okamoto S, Lipton SA (2013). Aberrant protein s-nitrosylation in neurodegenerative diseases. Neuron..

[3] Ahern GP, Klyachko VA, Jackson MB (2002). cGMP and S-nitrosylation: two routes for modulation of neuronal excitability by NO. Trends Neurosci..

[4] Lipton SA (1999). Neuronal protection and destruction by NO. Cell Death Differ..

[5] Ko AR, Hyun HW, Min SJ, Kim JE, Kang TC (2015). Endothelin-1 induces LIMK2-mediated programmed necrotic neuronal death independent of NOS activity. Mol Brain..

[6] Kim JE, Ryu HJ, Kang TC (2013). Status epilepticus induces vasogenic edema via tumor necrosis factor-α/ endothelin-1-mediated two different pathways. PLoS One.

[7] Jeon AR, Kim JE (2018). PDI knockdown inhibits seizure activity in acute seizure and chronic epilepsy rat models via S-nitrosylation-independent thiolation on NMDA receptor. Front Cell Neurosci.

[8] Lee DS, Kim JE (2018). PDI-mediated S-nitrosylation of DRP1 facilitates DRP1-S616 phosphorylation and mitochondrial fission in CA1 neurons. Cell Death Dis..

[9] Rassendren F, Buell GN, Virginio C, Collo G, North RA, Surprenant A (1997). The permeabilizing ATP receptor, P2X7. Cloning and expression of a human cDNA. J Biol Chem.

[10] Surprenant A, Rassendren F, Kawashima E, North RA, Buell G (1996). The cytolytic P_2Z_ receptor for extracellular ATP identified as a P_2X_ receptor (P2X_7_). Science.

[11] Adinolfi E, Pizzirani C, Idzko M, Panther E, Norgauer J, Di Virgilio F, Ferrari D (2005). P2X(7) receptor: death or life?. Purinergic Signal..

[12] Kim JE, Kang TC (2011). The P2X7 receptor-pannexin-1 complex decreases muscarinic acetylcholine receptor-mediated seizure susceptibility in mice. J Clin Invest..

[13] Kim JE, Ko AR, Hyun HW, Min SJ, Kang TC (2018). P2RX7-MAPK1/2-SP1 axis inhibits MTOR independent HSPB1-mediated astroglial autophagy. Cell Death Dis..

[14] Kim JE, Ryu HJ, Yeo SI, Kang TC (2011). P2X7 receptor differentially modulates astroglial apoptosis and clasmatodendrosis in the rat brain following status epilepticus. Hippocampus..

[15] Kim JE, Kim DS, Ryu HJ, Il Kim W, Kim MJ, Won Kim D, Young Choi S, Kang TC (2013). The effect of P2X7 receptor activation on nuclear factor-κB phosphorylation induced by status epilepticus in the rat hippocampus. Hippocampus.

[16] Choi HK, Ryu HJ, Kim JE, Jo SM, Choi HC, Song HK, Kang TC (2012). The roles of P2X7 receptor in regional-specific microglial responses in the rat brain following status epilepticus. Neurol Sci.

[17] Kim JE, Ryu HJ, Yeo SI, Kang TC (2010). P2X7 receptor regulates leukocyte infiltrations in rat frontoparietal cortex following status epilepticus. J Neuroinflammation..

[18] Egan TM, Cox JA, Voigt MM (2004). Molecular structure of P2X receptors. Curr Top Med Chem..

[19] Coddou C, Yan Z, Obsil T, Huidobro-Toro JP, Stojilkovic SS (2011). Activation and regulation of purinergic P2X receptor channels. Pharmacol Rev..

[20] Jindrichova M, Kuzyk P, Li S, Stojilkovic SS, Zemkova H (2012). Conserved ectodomain cysteines are essential for rat P2X7 receptor trafficking. Purinergic Signal..

[21] Gu BJ, Saunders BM, Petrou S, Wiley JS (2011). P2X(7) is a scavenger receptor for apoptotic cells in the absence of its ligand, extracellular ATP. J Immunol..

[22] Kim JY, Ko AR, Hyun HW, Min SJ, Kim JE (2017). PDI regulates seizure activity via NMDA receptor redox in rats. Sci Rep..

[23] Rigobello MP, Donella-Deana A, Cesaro L, Bindoli A (2001). Distribution of protein disulphide isomerase in rat liver mitochondria. Biochem J..

[24] Turano C, Coppari S, Altieri F, Ferraro A (2002). Proteins of the PDI family: unpredicted non-ER locations and functions. J Cell Physiol..

[25] Ramachandran N, Root P, Jiang XM, Hogg PJ, Mutus B (2001). Mechanism of transfer of NO from extracellular S-nitrosothiols into the cytosol by cell-surface protein disulfide isomerase. Proc Natl Acad Sci USA..

[26] Kim JE, Park H, Choi SH, Kong MJ, Kang TC (2019). Roscovitine attenuates microglia activation and monocyte Infiltration via p38 MAPK Inhibition in the rat frontoparietal cortex following status epilepticus. Cells..

[27] Kang TC, Kim DS, Kwak SE, Kim JE, Won MH, Kim DW, Choi SY, Kwon OS (2006). Epileptogenic roles of astroglial death and regeneration in the dentate gyrus of experimental temporal lobe epilepsy. Glia..

[28] Streit WJ, Walter SA, Pennell NA (1999). Reactive microgliosis. Prog Neurobiol..

[29] Christian F, Smith EL, Carmody RJ (2016). The regulation of NF-κB subunits by phosphorylation. Cells..

[30] Furusawa J, Funakoshi-Tago M, Tago K, Mashino T, Inoue H, Sonoda Y, Kasahara T (2009). Licochalcone A significantly suppresses LPS signaling pathway through the inhibition of NF-κB p65 phosphorylation at serine 276. Cell Signal..

[31] Zhao YL, Houk KN (2006). Thionitroxides, RSNHO*: the structure of the SNO moiety in “S-Nitrosohemoglobin”, a possible NO reservoir and transporter. J Am Chem Soc.

[32] Ko AR, Kim JY, Hyun HW, Kim JE (2015). Endoplasmic reticulum (ER) stress protein responses in relation to spatio-temporal dynamics of astroglial responses to status epilepticus in rats. Neuroscience..

[33] Caggiano AO, Kraig RP (1996). Eicosanoids and nitric oxide influence induction of reactive gliosis from spreading depression in microglia but not astrocytes. J Comp Neurol.

[34] Deep SN, Baitharu I, Sharma A, Gurjar AK, Prasad D, Singh SB (2016). Neuroprotective role of L-NG-nitroarginine methyl ester (L-NAME) against chronic hypobaric hypoxia with crowding stress (CHC) induced depression-like behaviour. PLoS One..

[35] Lipton SA, Choi YB, Takahashi T, Zhang D, Li W, Godzik A, Bankston LA (2002). Cysteine regulation of protein function—as exemplified by NMDA-receptor modulation. Trends Neurosci..

[36] Stamler JS, Toone EJ (2002). The decomposition of thionitrites. Curr Opin Chem Biol..

[37] Cho DH, Nakamura T, Fang J, Cieplak P, Godzik A, Gu Z, Lipton SA (2009). S-Nitrosylation of Drp1 mediates beta-amyloid-related mitochondrial fission and neuronal injury. Science..

[38] Sim JA, Young MT, Sung HY, North RA, Surprenant A (2004). Reanalysis of P2X7 receptor expression in rodent brain. J Neurosci..

[39] Panenka W, Jijon H, Herx LM, Armstrong JN, Feighan D, Wei T, Yong VW, Ransohoff RM, MacVicar BA (2001). P2X7-like receptor activation in astrocytes increases chemokine monocyte chemoattractant protein-1 expression via mitogen-activated protein kinase. J Neurosci..

[40] Kukley M, Barden JA, Steinhäuser C, Jabs R (2001). Distribution of P2X receptors on astrocytes in juvenile rat hippocampus. Glia..

[41] Sperlágh B, Vizi ES, Wirkner K, Illes P (2006). P2X7 receptors in the nervous system. Prog Neurobiol..

[42] Ozaki T, Yamashita T, Ishiguro S (1783). ERp57-associated mitochondrial μ-calpain truncates apoptosis-inducing factor. Biochim Biophys Acta..

[43] Uehara T, Nakamura T, Yao D, Shi ZQ, Gu Z, Ma Y, Masliah E, Nomura Y, Lipton SA (2006). S-Nitrosylated protein-disulphide isomerase links protein misfolding to neurodegeneration. Nature..

[44] Ho GP, Selvakumar B, Mukai J, Hester LD, Wang Y, Gogos JA, Snyder SH (2011). S-Nitrosylation and S-palmitoylation reciprocally regulate synaptic targeting of PSD-95. Neuron..

[45] Gonnord P, Delarasse C, Auger R, Benihoud K, Prigent M, Cuif MH, Lamaze C, Kanellopoulos JM (2009). Palmitoylation of the P2X7 receptor, an ATP-gated channel, controls its expression and association with lipid rafts. FASEB J..

[46] Jeon GS, Nakamura T, Lee JS, Choi WJ, Ahn SW, Lee KW, Sung JJ, Lipton SA (2014). Potential effect of S-nitrosylated protein disulfide isomerase on mutant SOD1 aggregation and neuronal cell death in amyotrophic lateral sclerosis. Mol Neurobiol..

[47] Caseley EA, Muench SP, Jiang LH (2017). Conformational changes during human P2X7 receptor activation examined by structural modelling and cysteine-based cross-linking studies. Purinergic Signal..

[48] Kim JE, Ryu HJ, Kang TC (2011). P2X7 receptor activation ameliorates CA3 neuronal damage via a tumor necrosis factor-α-mediated pathway in the rat hippocampus following status epilepticus. J Neuroinflammation..

[49] Di Virgilio F, Dal Ben D, Sarti AC, Giuliani AL, Falzoni S (2017). The P2X7 receptor in infection and inflammation. Immunity..

[50] Ousingsawat J, Wanitchakool P, Schreiber R, Kunzelmann K (2018). Contribution of TMEM16F to pyroptotic cell death. Cell Death Dis..

[51] Ding J, Wang K, Liu W, She Y, Sun Q, Shi J, Sun H, Wang DC, Shao F (2016). Pore-forming activity and structural autoinhibition of the gasdermin family. Nature..

[52] Liu X, Zhang Z, Ruan J, Pan Y, Magupalli VG, Wu H, Lieberman J (2016). Inflammasome-activated gasdermin D causes pyroptosis by forming membrane pores. Nature..

