# Supplementary material for: Protein disulfide isomerase-mediated S-nitrosylation facilitates surface expression of P2X7 receptor following status epilepticus
Source: J Neuroinflammation. 2021 Jan 6;18:14. doi: 10.1186/s12974-020-02058-y (PMC7788848; doi:10.1186/s12974-020-02058-y)
Supplement: Supplementary file 1 — Additional file 1. [file 12974_2020_2058_MOESM1_ESM.pdf]

Supplementary information

Protein disulfide isomerase-mediated *S*-nitrosylation facilitates surface expression of P2X7 receptor following status epilepticus

Duk-Shin Lee[1] and Ji-Eun Kim[1,*]

[1]Department of Anatomy and Neurobiology, Institute of Epilepsy Research, College of Medicine, Hallym University, Chuncheon 24252, South Korea

Running title: PDI-mediated S-nitrosylation of P2X7 receptor

* Correspondence to: J.-E Kim, Department of Anatomy and Neurobiology, College of Medicine, Hallym University, Chunchon, Kangwon-Do 24252, South Korea; Tel: +82-33-248-2522; Fax: +82-33-248-2525; E-mail: jieunkim@hallym.ac.kr

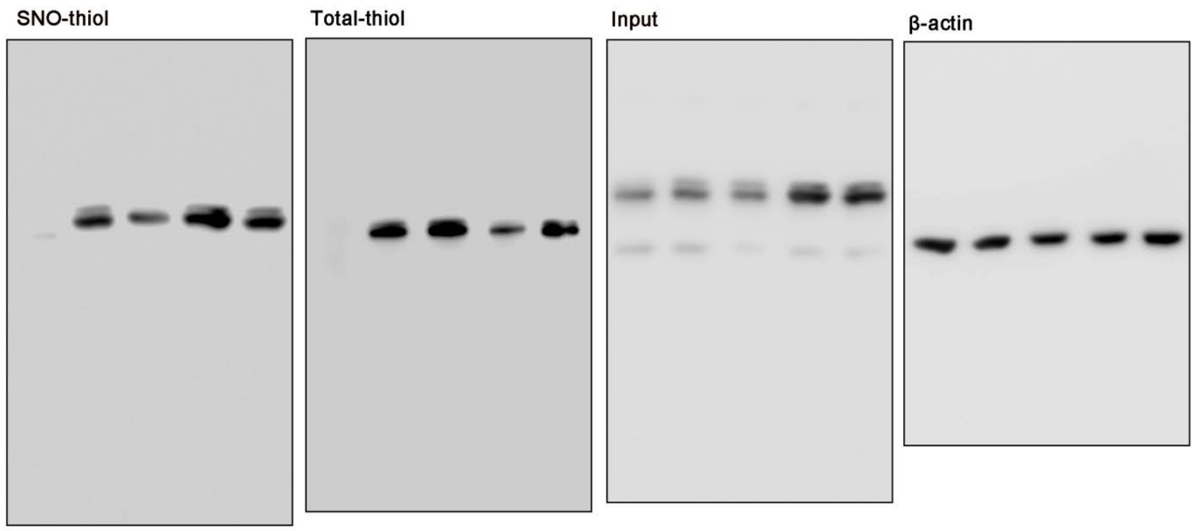

Supplementary Figure 1. Full-length gel images of western blot data in Fig. 2A.

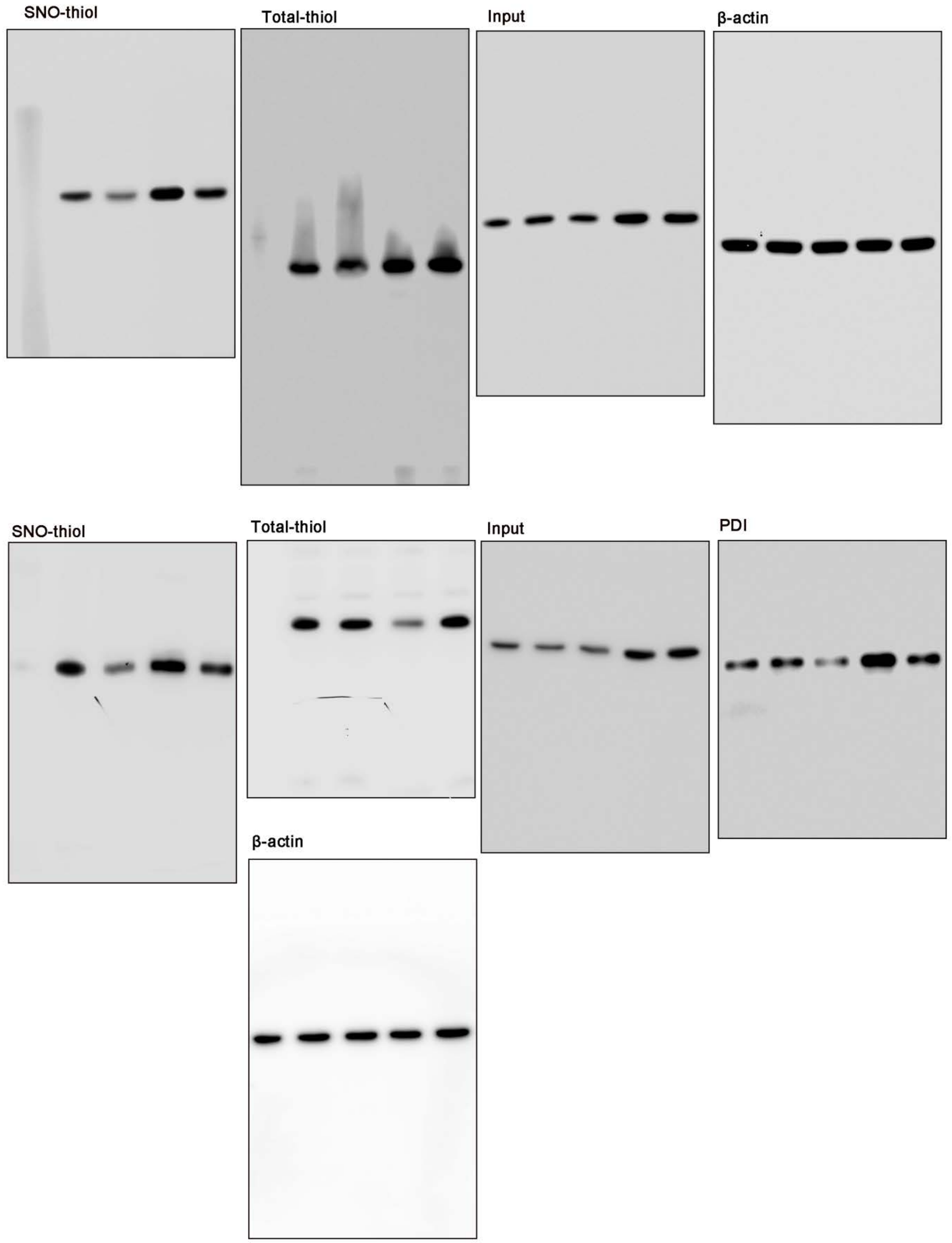

Supplementary Figure 2. Full-length gel images of western blot data in Fig. 5A and E.

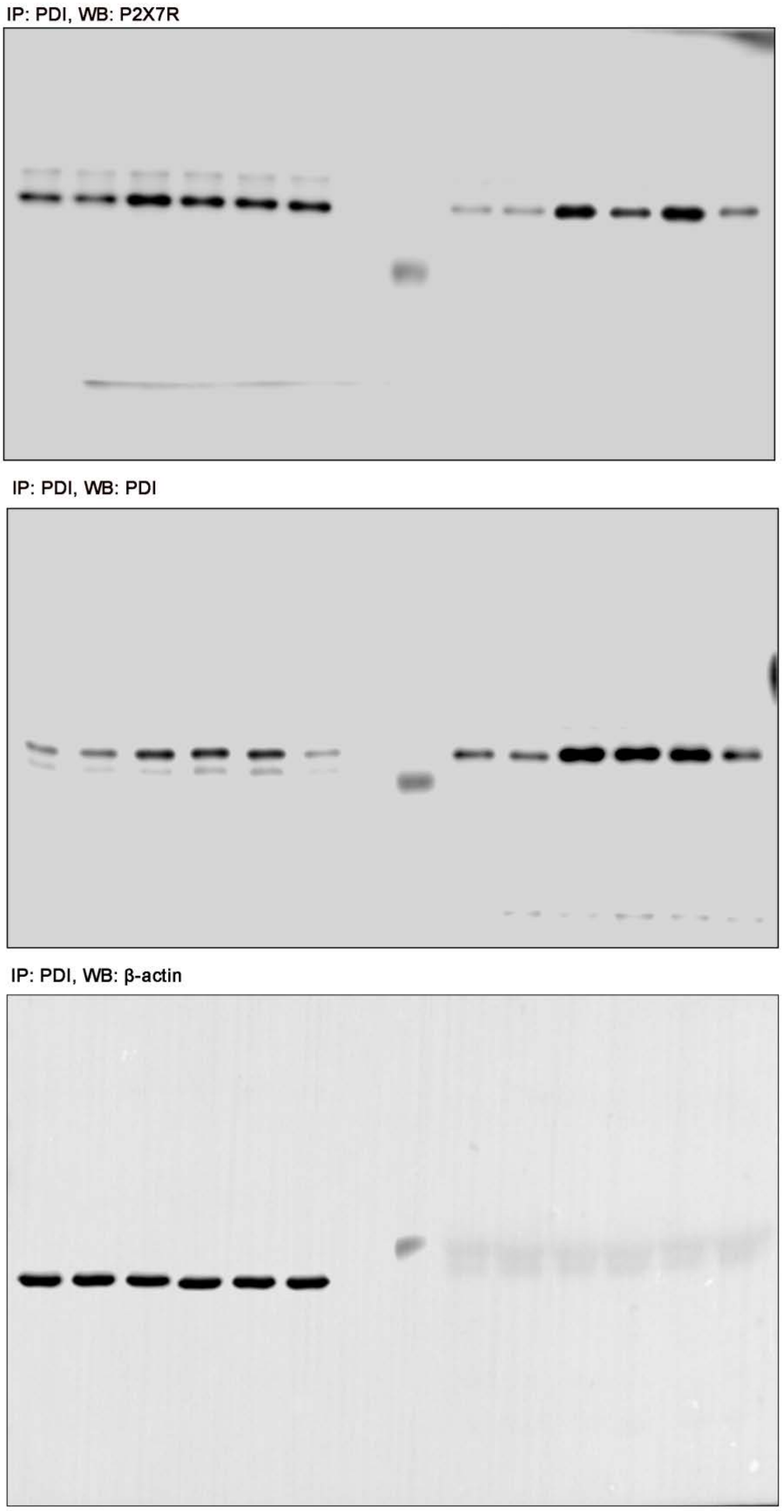

Supplementary Figure 3. Full-length gel images of western blot data in Fig. 5C.

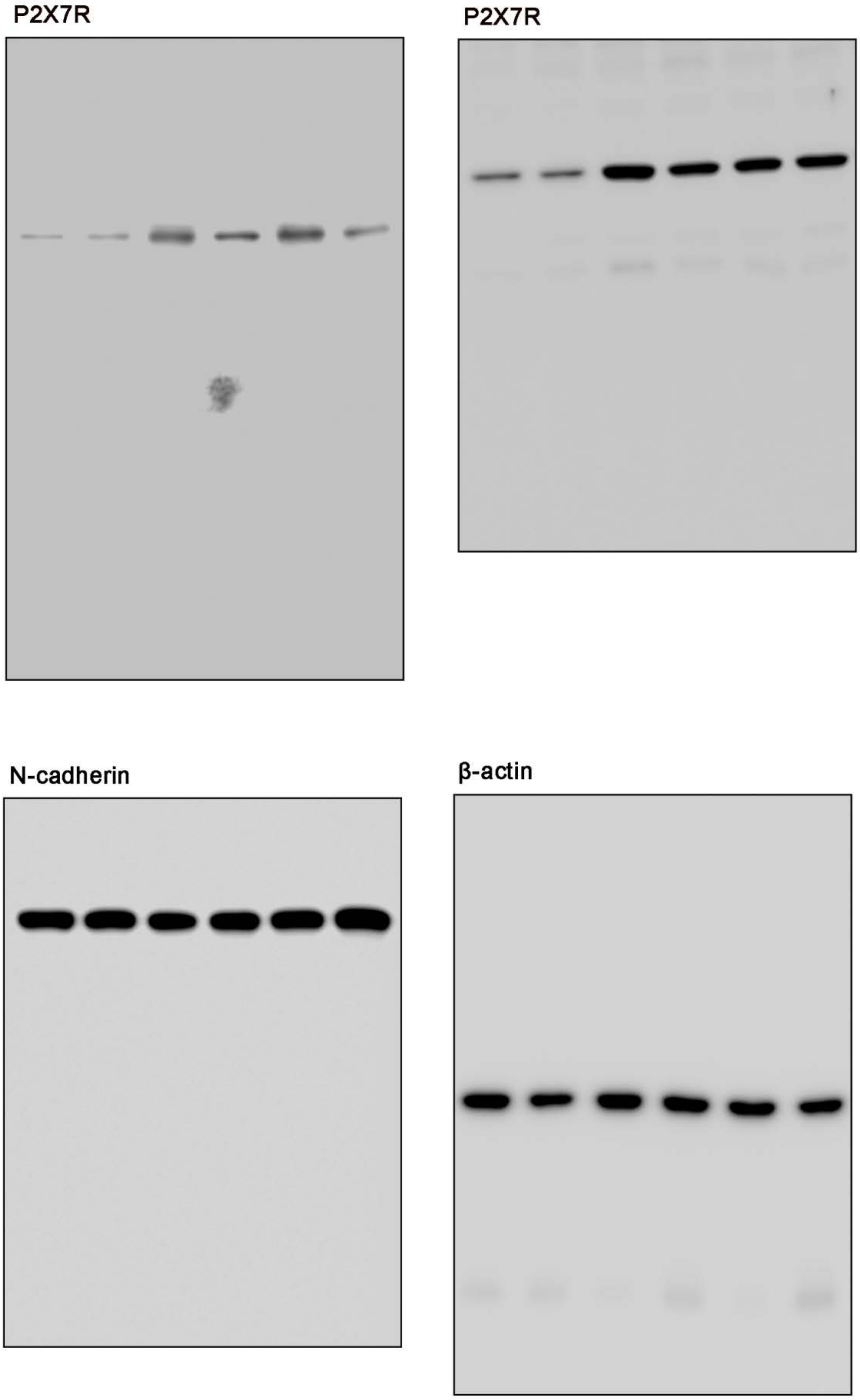

Supplementary Figure 4. Full-length gel images of western blot data in Fig. 7A.
